# Supplementary material for: 5-Aminolevulinic Acid (5-ALA)-Induced Protoporphyrin IX Fluorescence by Glioma Cells—A Fluorescence Microscopy Clinical Study
Source: Cancers (Basel). 2022 Jun 8;14(12):2844. doi: 10.3390/cancers14122844 (PMC9221265; doi:10.3390/cancers14122844)
Supplement: Supplementary file 1 [file cancers-14-02844-s001.zip › Supplementary Figure S1.pdf]

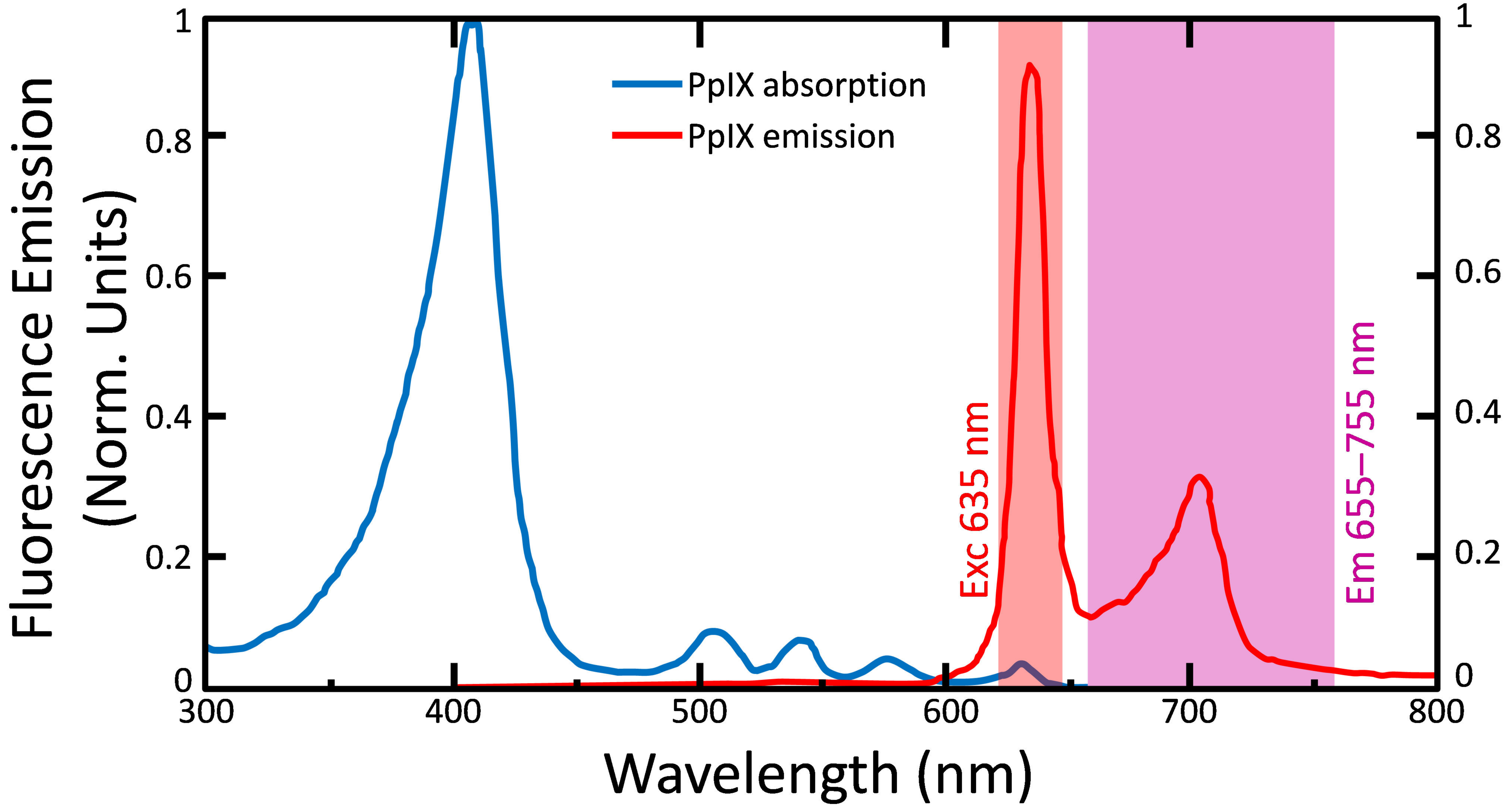

Supplementary Figure S1. Graph showing the PpIX absorption (excitation) and normalized emission spectra. Red and purple shaded areas correspond to the portion of PpIX excitation (Exc: 625 nm) and emission (Em: 655–755 nm) spectrum used to detect the 5-ALA signal. (Modified from Roberts, D.W.; et al., *J Neurosurg* **2018,** 128, 1690–1697 [17])
