# Supplementary material for: 5-Aminolevulinic Acid (5-ALA)-Induced Protoporphyrin IX Fluorescence by Glioma Cells—A Fluorescence Microscopy Clinical Study
Source: Cancers (Basel). 2022 Jun 8;14(12):2844. doi: 10.3390/cancers14122844 (PMC9221265; doi:10.3390/cancers14122844)
Supplement: Supplementary file 1 [file cancers-14-02844-s001.zip › Supplementary Figure S2.pdf]

Supplementary Figure S2

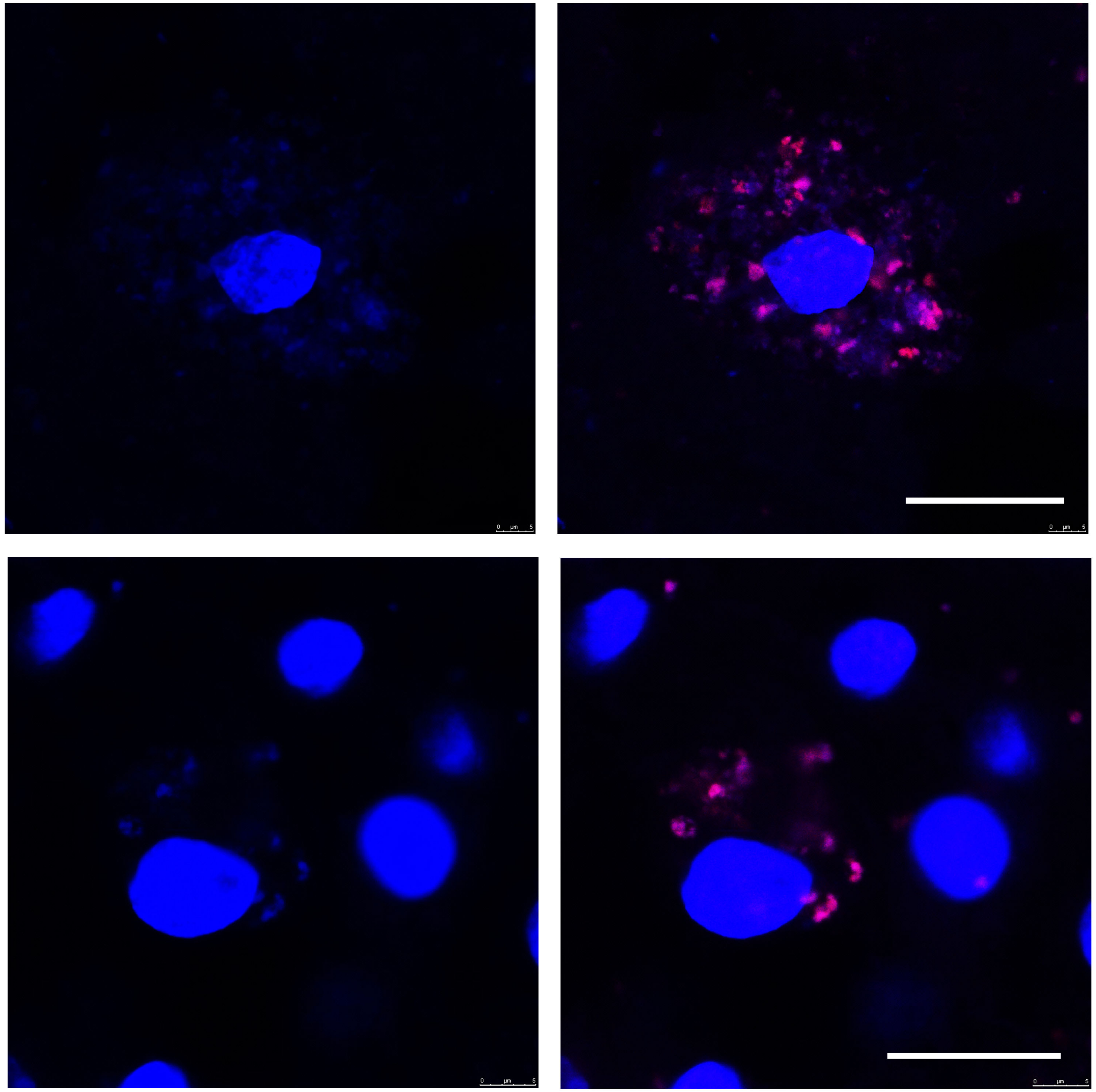

Supplementary Figure S2 . Two-channel acquisition exciting a GBM sample (*Case #26 in Table 1*) with a 405 nm diode laser and acquiring the emission at wavelenghts ranging from 416 to 480 nm (*left panels*) and from 630 to 700 nm (*right panels*). As for the excitation wavelenght of 635 nm, 5-ALA gave a dotted signal in the cell cytoplasm. Scale bars, 20 μm.
