# Supplementary material for: 5-Aminolevulinic Acid (5-ALA)-Induced Protoporphyrin IX Fluorescence by Glioma Cells—A Fluorescence Microscopy Clinical Study
Source: Cancers (Basel). 2022 Jun 8;14(12):2844. doi: 10.3390/cancers14122844 (PMC9221265; doi:10.3390/cancers14122844)
Supplement: Supplementary file 1 [file cancers-14-02844-s001.zip › Supplementary Figure S3.pdf]

Supplementary Figure S3

A

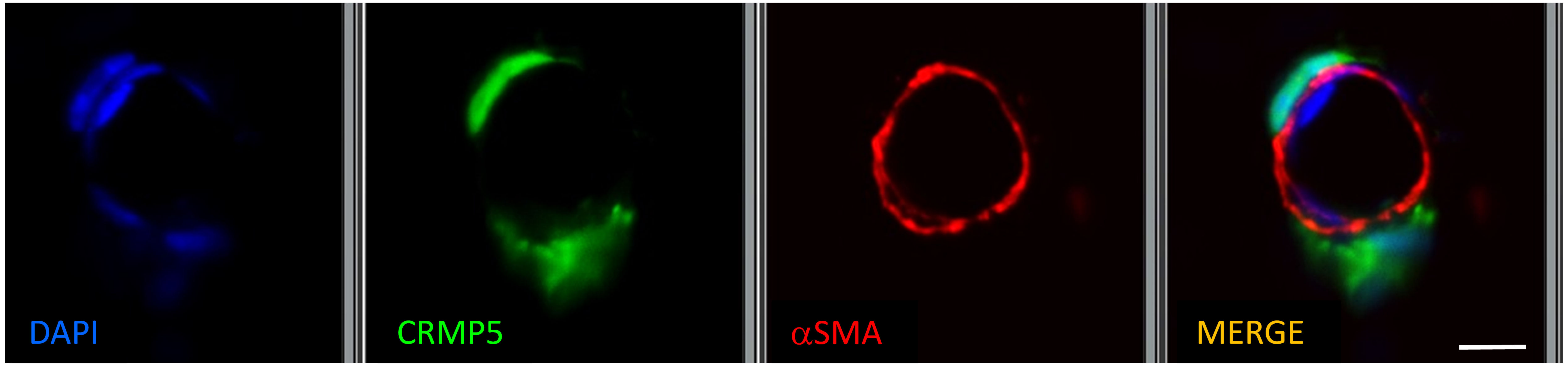

B

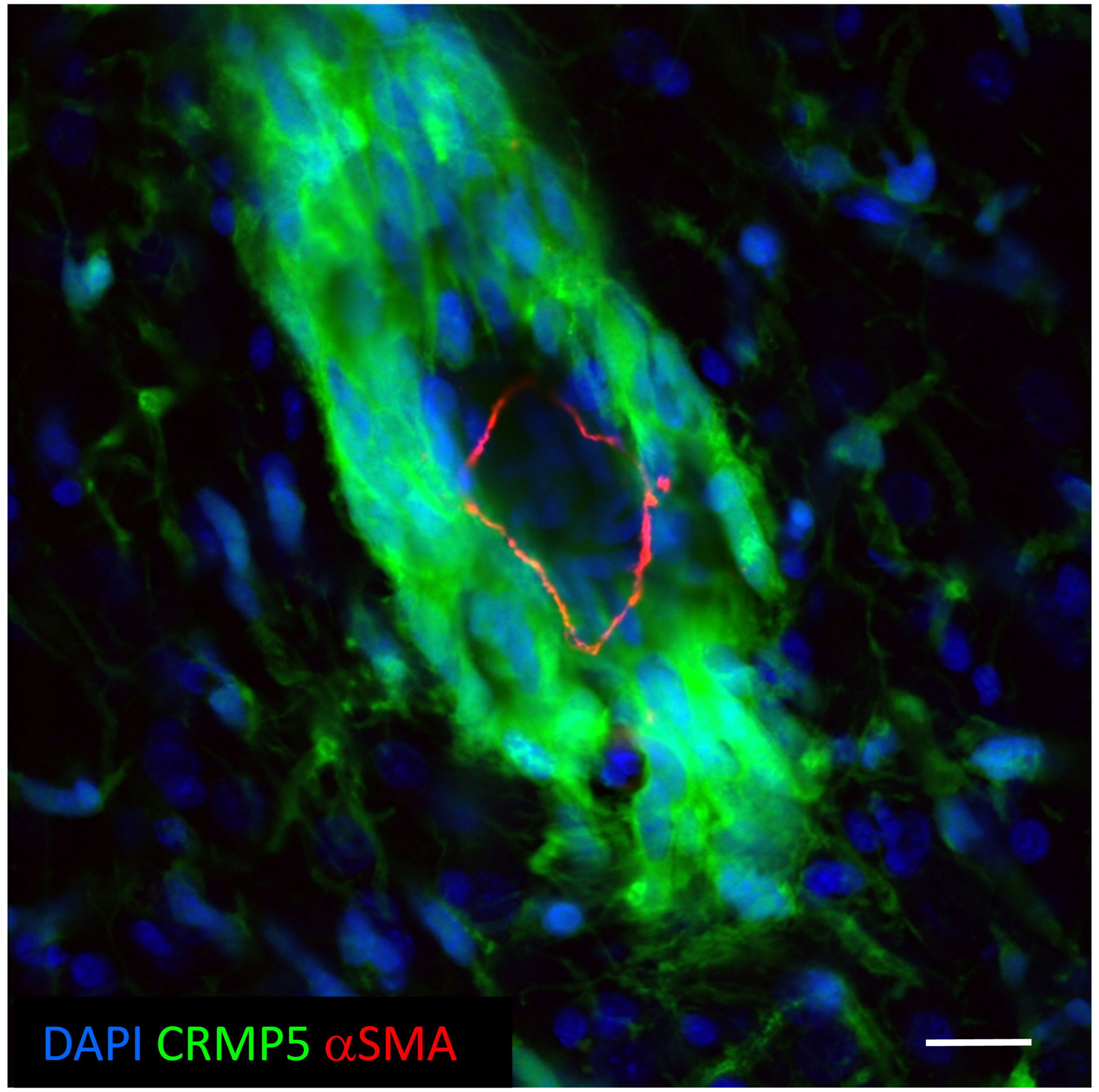

Supplementary Figure S3.

A. Confocal image showing perivascular infiltration by tumor cells (*green*). Activated pericytes are stained in *red*.

B. Confocal image showing a brain vessel with thickned wall due to multilayered tumor cells (*green*) invading along the perivascular space. Activated pericytes (*red*) contribute to the vessel wall with a thin layer. Scale bars, 20 μm
