# Supplementary material for: 5-Aminolevulinic Acid (5-ALA)-Induced Protoporphyrin IX Fluorescence by Glioma Cells—A Fluorescence Microscopy Clinical Study
Source: Cancers (Basel). 2022 Jun 8;14(12):2844. doi: 10.3390/cancers14122844 (PMC9221265; doi:10.3390/cancers14122844)
Supplement: Supplementary file 1 [file cancers-14-02844-s001.zip › Supplementary Figure S4.pdf]

Supplementary Figure S4

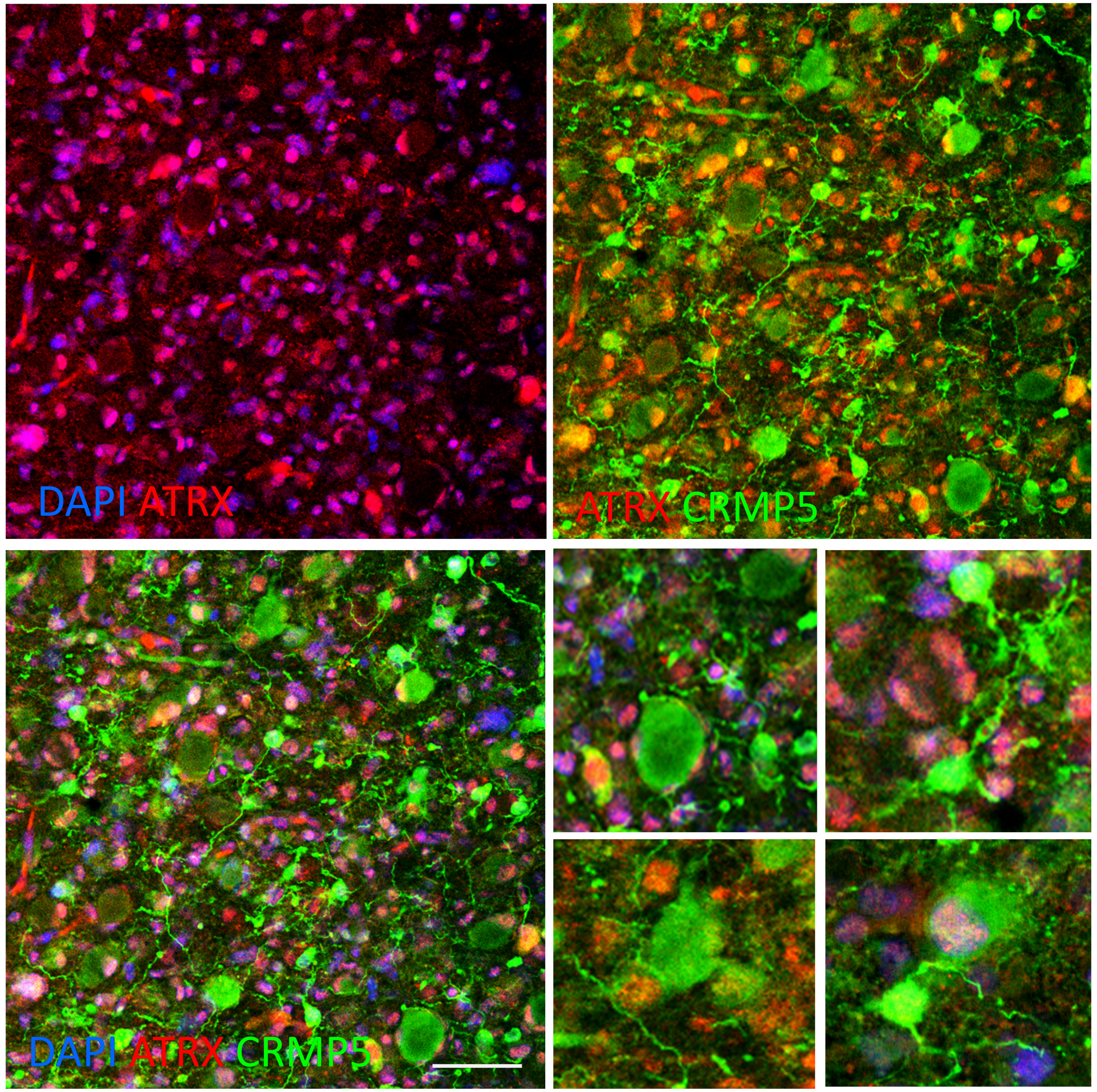

Supplementary Figure S4. Double immunofluorescence microscopy of a giant cell GBM with anti-ATRX and anti-CRMP5 antibodies. The cell nuclei are counterstained with DAPI. In general, there was a good relationship between CRMP5 expression and loss or hypo-expression of ATRX by the tumor cells. Several CRMP5 expressing tumor cells have lost ATRX expression in their nuclei (*insets*). Scale bar, 60 μm.
